# Examining the Impact of Mesoporous Zinc Oxide Nanoparticle Inclusion on the Surface Microhardness and Roughness of Resin-Modified Glass Ionomer Cement: An In Vitro Analysis

**DOI:** 10.1155/ijod/5491727

**Published:** 2025-09-29

**Authors:** Zahra Jowkar, Zahra Kazemi, Fereshteh Shafiei, Seyed Ahmadreza Hamidi, Ali Moaddeli

**Affiliations:** ^1^Oral and Dental Disease Research Center, Department of Operative Dentistry, School of Dentistry, Shiraz University of Medical Sciences, Shiraz, Iran; ^2^Department of Operative Dentistry, School of Dentistry, Shiraz University of Medical Sciences, Shiraz, Iran; ^3^Department of Oral and Maxillofacial Surgery, School of Dentistry, Shiraz University of Medical Sciences, Shiraz, Iran; ^4^Legal Medicine Research Center, Legal Medicine Organization, Tehran, Iran

**Keywords:** mesoporous, resin-modified glass ionomer cement, roughness, surface microhardness, zinc oxide nanoparticles

## Abstract

**Aim:**

Addressing the need for enhanced antibacterial properties in dental materials, this study investigated the impact of integrating zinc oxide nanoparticles (ZnO NPs) and mesoporous ZnO NPs into resin-modified glass ionomer cement (RMGIC) on microhardness and surface roughness.

**Materials and Methods:**

Seventy disk-shaped RMGIC specimens were allocated to seven experimental groups: RMGIC (control), RMGIC with 3 wt.% ZnO NPs, 5 wt.% ZnO NPs, 7 wt.% ZnO NPs, 3 wt.% mesoporous ZnO NPs, 5 wt.% mesoporous ZnO NPs, and 7 wt.% mesoporous ZnO NPs. Surface roughness and Vickers microhardness were quantified using a surface profilometer and Vickers microhardness tester, respectively. Statistical analysis was carried out with a significance level set at *p* < 0.05.

**Result:**

Incorporating 5 wt.% of ZnO NPs or mesoporous ZnO NPs into the RMGIC yielded the highest microhardness values, while the control group exhibited the lowest microhardness values. Notably, the microhardness values of RMGIC with 3 and 5 wt.% ZnO NPs or mesoporous ZnO NPs were significantly higher than those of the 7 wt.% concentration. Regarding surface roughness, the control group displayed the highest roughness value, while RMGIC with 5 wt.% mesoporous ZnO NPs exhibited the lowest roughness values. Therefore, incorporating up to 5 wt.% ZnO NPs or mesoporous ZnO NPs led to decreased roughness values, with a notable increase observed at the 7 wt.% concentration, albeit still lower than the control group's roughness values.

**Conclusion:**

Incorporating 5 wt.% ZnO NPs or mesoporous ZnO NPs resulted in significantly enhanced microhardness values compared to the control group and the 7 wt.% concentration. The introduction of up to 5 wt.% NPs led to reduced surface roughness, with the 7 wt.% concentration showing a slight increase in roughness. These findings highlight the importance of optimizing NP concentrations, particularly mesoporous NPs, in RMGIC to enhance mechanical properties, offering valuable insights for the development of dental materials with improved performance characteristics.

## 1. Introduction

Numerous strategies have been investigated to improve the mechanical properties and adhesive performance of restorative materials on various dental substrates [1–3]. Among these, glass ionomer cements (GICs) are widely used in restorative dentistry due to their inherent fluoride release and ability to chemically bond to tooth structure. GICs serve multiple clinical roles, including as cavity bases, luting agents, and sealants [4]. GICs are highly valued for preventing cavities in high-risk patients because of their ability to release fluoride [4, 5]. They offer several advantages, such as continuous fluoride release for cavity prevention, bonding chemically to tooth structures, gentle pulp response, thermal compatibility with teeth, and a natural appearance matching tooth color [6]. However, drawbacks of GICs include weaker mechanical properties than composite resins, limited translucency affecting aesthetics, sensitivity to moisture during setting limiting their use to low-stress areas, along with deficiencies in wear and abrasion resistance [6, 7].

A range of materials have been integrated into GICs to boost their mechanical attributes [6, 8–10]. Resin-modified GICs (RMGICs) were created to improve the physical and mechanical properties of conventional GICs (CGICs), resulting in increased compressive and flexural strength due to the incorporation of resin monomers [11]. Additionally, RMGICs enable partial polymerization through light activation, granting clinicians the ability to regulate the setting process [11].

Maintaining surface texture and quality is crucial for dental materials, helping to minimize negative biological reactions and bacterial attachment [5]. Smoothing surfaces can reduce biofilm, a significant contributor to dental problems like cavities and gum disease [5]. A polished surface promotes mechanical strength, aesthetics, optical compatibility with enamel, and glossiness, preventing staining and imperfections [5]. Microhardness, a key mechanical property indicating resistance to deformation, significantly influences the effectiveness of dental restorations [5]. Improving the surface characteristics of GICs is essential for addressing wear resistance and microhardness concerns. Research underlines the direct link between microhardness and a material's ability to endure mechanical wear, underscoring the need to increase surface hardness to minimize wear [7]. Strategies like protective coatings have been proposed to enhance GICs' wear resistance and microhardness. Despite attempts to enhance wear resistance with resin coatings, studies have shown limited short- to medium-term efficacy in guarding against abrasive wear [7, 12]. The durability of existing resin coatings designed to shield GIC surfaces has been noted to be limited [7]. Moreover, Valeri et al. [13] reported that certain glass-ionomer-containing restorative materials, such as Equia Forte HT, exhibited significantly higher in vitro wear compared to a resin composite. Consequently, ongoing endeavors aim to refine the surface properties of GICs to combat these challenges, with approaches including adjusting compositions or integrating fillers into the GIC structure to heighten surface microhardness [9, 12, 14].

In restorative dentistry, managing bacterial colonization on tooth walls after caries removal during tooth preparation is crucial [14, 15]. The presence of bacteria poses a threat to the durability of dental restorations. Incorporating antibacterial agents into restorative materials is a strategy to counteract bacterial invasion and growth, and reduce the likelihood of recurrent caries [14, 15].

Zinc oxide (ZnO) is renowned for its antimicrobial attributes, widely utilized in diverse dental materials like gutta-percha, endodontic sealers, zinc polycarboxylate cement, and ZnO eugenol [15]. Furthermore, ZnO is cost-effective, chemically stable, and nontoxic [15]. The effectiveness of ZnO in combating bacteria stems from its affinity towards bacterial cells, altering their membrane function and enzymatic processes [15]. Over the last two decades, researchers have delved into synthesizing and applying nanoscale ZnO particles to enhance their antibacterial efficacy [15, 16]. Nanoparticles (NPs) have gained attention in dentistry due to their potent antibacterial properties, penetrating dentinal tubules more effectively than traditional antibacterial solutions. ZnO NPs have demonstrated significant antibacterial effects against various dental plaque bacteria, including Lactobacillus and *S. mutans* [17]. They have been integrated into dental materials as both reinforcing agents and antibacterial components to combat biofilm adhesion [18]. Furthermore, ZnO NPs have been utilized in enamel and dentin pretreatments before composite bonding procedures, improving bond strength without compromise [19]. Studies have shown that using ZnO NPs as a final irrigation solution in root canal therapy enhances the fracture resistance of treated roots and improves bond strength values of GIC to dentin following dentin pretreatment [8, 16].

Recent attention in the medical and dental realms has shifted towards mesoporous materials, characterized by pore sizes ranging from 2 to 50 nm [3, 20]. These materials offer adjustable mesoporous pore size, high specific surface area, nontoxicity, and strong biological compatibility [21]. Their pores can be readily modified and functionalized, while their synthesis allows for optimization of composition, structure, and pore size [21]. Mesoporous ZnO NPs, known for their significant surface area, porosity volume, crystallinity, and antibacterial potential, are emerging as promising candidates for multifunctional therapeutic applications [22].

The imperative to develop novel biocompatible antibacterial fillers for integration into restorative materials, such as RMGICs, is crucial in modern dentistry. Nano-sized materials like mesoporous ZnO NPs offer significantly improved antibacterial properties compared to their solid bulk counterparts due to their elevated surface area-to-volume ratio [22]. Therefore, they represent promising nanofillers for incorporation into restorative materials like RMGIC. However, the integration of these nanofillers alongside restorative materials to enhance antibacterial efficacy must not compromise the physical and mechanical attributes of the restorative materials. Hence, it is essential to first evaluate this aspect. The uncertainty surrounding the effects of introducing mesoporous ZnO NPs into RMGIC on its physical and mechanical properties necessitates further investigation. Previous studies have not delved into how the inclusion of mesoporous ZnO NPs and ZnO NPs impacts the surface microhardness and roughness of RMGIC. This study aimed to investigate and compare the microhardness and surface roughness of RMGIC with and without the inclusion of mesoporous ZnO NPs and ZnO NPs. The null hypothesis suggested that there would be no significant difference in microhardness and surface roughness between RMGIC samples with and without the addition of mesoporous ZnO NPs and ZnO NPs.

## 2. Materials and Methods

**Figure 1:**
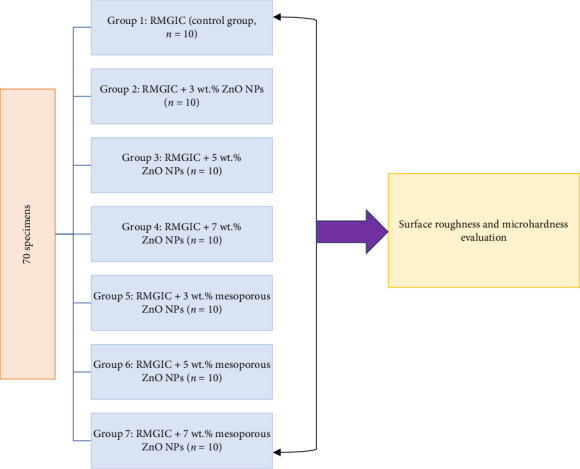
Schematic diagram of the experimental study design. RMGIC, resin-modified glass ionomer cement; ZnO NPs, zinc oxide nanoparticles.

The research protocol was approved by the Research and Ethics Committee of Shiraz University of Medical Sciences under Protocol # IR.SUMS.DENTAL.REC.1403.014. ZnO NPs were procured from Asepe Nanomaterials Company, Tabriz, Iran, while the preparation and characterization of mesoporous ZnO NPs followed a method outlined in an earlier investigation [23]. The synthesized mesoporous ZnO NPs exhibited a quasi-spherical morphology with an average particle size of 10–15 nm as observed via transmission electron microscopy (TEM), while field emission scanning electron microscopy (FESEM) showed a broader aggregated particle size distribution of 70–100 nm. Brunauer–Emmett–Teller (BET) analysis confirmed a mesoporous structure with a specific surface area of 5 m^2^/g, and Barrett–Joyner–Halenda (BJH) analysis indicated two dominant pore sizes of 8.5 and 27 nm. X-ray diffraction (XRD) revealed a highly crystalline hexagonal wurtzite structure, and dynamic light scattering (DLS) reported an average hydrodynamic particle size of 115.27 nm. These characteristics confirmed the mesoporous, crystalline, and nanoscale nature of the ZnO particles used in this study. The illustration of the experimental layout is presented in Figure 1.

### 2.1. Sample Preparation

In this experimental study, seven batches of disk-shaped samples were prepared, each consisting of 10 specimens measuring 2 mm in height and 6 mm in diameter. The sample size for the study was determined using G Power software (G^*⁣*^*∗*^^Power 3.1 software; Heinrich Heine University, Dusseldorf, Germany) based on prior literature, considering a power level of 80% and a significance level of 0.05 [24]. The effect size (*f*) was estimated at approximately 1.30, with a type I error (*α*) of 0.05 and a type II error (*β*) of 0.2, requiring at least nine specimens per subgroup. Consequently, 10 specimens were selected for each experimental group. Prior to the main experiment, a pilot study was conducted to determine the appropriate range of NP concentrations. In this preliminary assessment, mesoporous and conventional ZnO NPs were incorporated into RMGIC at concentrations ranging from 1 to 10 wt.% (*n* = 3 per concentration). The mechanical properties of the resulting specimens were evaluated in terms of surface microhardness and roughness. It was observed that improvements in these properties occurred with concentrations up to 7 wt.%. However, at concentrations of 8 wt.% and above, NP agglomeration became evident, accompanied by a reduction in Vickers hardness and an increase in surface irregularities. Based on these findings, concentrations of 3, 5, and 7 wt.% were selected for the main study, as they were considered optimal for enhancing performance without compromising structural integrity.

In crafting the samples, a cylindrical brass mold with a hollow center was utilized. Using a vibrating apparatus (Denstar-500; Denstar, Daegu, South Korea), the molds were filled for 15 s per sample to prevent air bubble formation. Seventy samples were carefully prepared and then divided into separate groups, each consisting of 10 samples, as outlined:

  Group 1: RMGIC samples (control group).
  Group 2: RMGIC samples with 3 wt.% ZnO NPs.
  Group 3: RMGIC samples with 5 wt.% ZnO NPs.
  Group 4: RMGIC samples with 7 wt.% ZnO NPs.
  Group 5: RMGIC samples with 3 wt.% mesoporous ZnO NPs.
  Group 6: RMGIC samples with 5 wt.% mesoporous ZnO NPs.
  Group 7: RMGIC samples with 7 wt.% mesoporous ZnO NPs.

In the control group (Group 1, *n* = 10), the disks were produced by mixing one scoop of a control RMGIC powder (Fuji II LC Gold A2; GC, Tokyo, Japan) with two liquid drops for 25 s following the manufacturer's instructions, maintaining a powder-to-liquid ratio of 3.2:1 by weight. The molds were slightly overfilled, and a Mylar matrix strip was applied to the top surface. Subsequently, the top surface of each sample was subjected to a 40 s light-curing process using an LED light-curing device (Blue Lex LD-105; Monitex, Taipei, Taiwan) with a light intensity of 1200 mW/cm^2^, positioned 1 mm from the sample's top surface as per the manufacturer's guidelines. In Group 2 (RMGIC + 3 wt.% ZnO NPs, *n* = 10), the samples were composed of 97 wt.% RMGIC and 3 wt.% ZnO NPs. Group 3 (RMGIC + 5 wt.% ZnO NPs, *n* = 10) included specimens with 95 wt.% RMGIC and 5 wt.% ZnO NPs, while Group 4 (RMGIC + 7 wt.% ZnO NPs, *n* = 10) consisted of samples with 93 wt.% RMGIC and 7 wt.% ZnO NPs. Furthermore, in Group 5 (RMGIC + 3 wt.% mesoporous ZnO NPs, *n* = 10), the specimens were formulated with 97 wt.% RMGIC and 3 wt.% mesoporous ZnO NPs. For Group 6 (RMGIC + 5 wt.% mesoporous ZnO NPs, *n* = 10), the samples contained 95 wt.% RMGIC and 5 wt.% mesoporous ZnO NPs, and Group 7 (RMGIC + 7 wt.% mesoporous ZnO NPs, *n* = 10) comprised specimens with 93 wt.% RMGIC and 7 wt.% mesoporous ZnO NPs. Subsequently, the samples in groups 2–6 were prepared and evaluated following the same procedure as delineated for Group 1.

**Figure 2:**
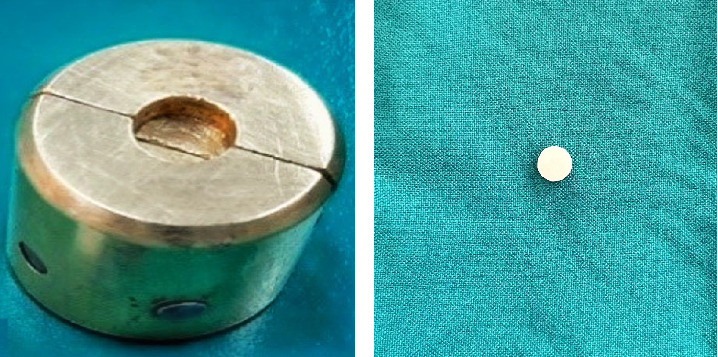
(a) Brass mold used for specimen preparation. (b) Prepared specimen for surface microhardness and roughness testing.

After the specimens had completely solidified, they were removed from the mold and covered with a thin coat of copal varnish (Kimia Varnish; Kimia, Tehran, Iran) for moisture protection. Subsequently, all samples were placed in a chamber with 100% humidity at 37°C for 24 h. They were then carefully polished using a low-speed handpiece (Ti-Max X25L; NSK, Tochigi, Japan) and a series of aluminum oxide polishing discs (TOR VM, Moscow, Russia) with grit sizes of coarse (100 µm), medium (40 µm), fine (24 µm), and superfine (8 µm). Discs were used consecutively from coarse to superfine, with each grit applied to the top surface of each specimen for 60 s under consistent pressure and speed. To eliminate any surface contaminants, the specimens were rinsed in distilled water in an ultrasonic bath (Easyclean; Renfert, Hilzingen, Germany) for 1 min. After cleaning, the samples were stored in an incubator at 37°C with 100% humidity for 24 h to allow for full maturation. Following this storage period, the specimens underwent assessments for both microhardness and surface roughness. An illustrated example of a specimen is presented in Figure 2.

**Figure 3:**
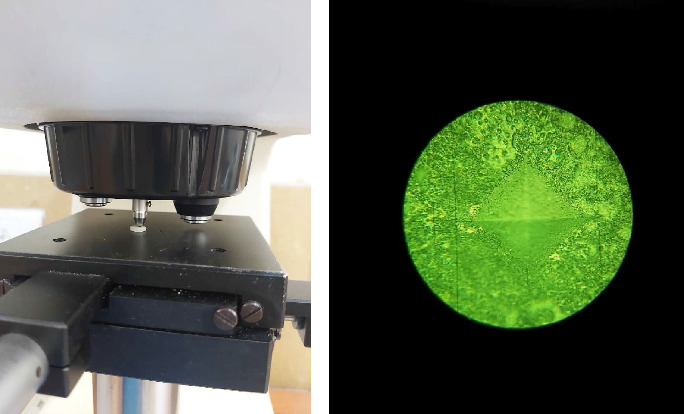
(a) Microhardness assessment of an experimental disk using a digital Vickers microhardness tester. (b) Indentation marks on the specimen surface.

Surface roughness (*R*_*a*_, μm) evaluations were carried out at three specific points on the upper surface of each specimen [9]. A profilometer (Rugosurf 20; TESA Technology, Renens, Switzerland), calibrated before the assessments, was used to measure *R*_*a*_. The stylus of the device made contact with the sample's surface, moving across the designated areas to capture surface profile details. Through precise assessment of surface irregularities, the profilometer aided in determining *R*_*a*_ values. To ensure measurement accuracy, three readings were taken at each specified location on the sample's surface. Subsequently, the average of these readings was calculated to determine the representative *R*_*a*_ value for each sample. To assess the microhardness of the top surfaces of the samples at three points, a digital Vickers microhardness tester (HXD-1000TMC; Taiming Optical Instrument, Shanghai, China) applying a force of 300 g for 15 s was employed (Figure 3a) [9]. The Vickers hardness number (VHN) was tested for each sample on three points with a distance of at least 1 mm from each other, and the average was documented as the average mean VHN value. The indentation surface is depicted in Figure 3b.

The statistical analyses were processed using IBM SPSS software version 20.0 for Windows (IBM SPSS software; SPSS, Chicago, IL, USA). Kolmogorov–Smirnov test was used to assess the normality of the data. Subsequently, the effects of NP integration on the surface roughness and microhardness were examined using a one-way ANOVA test followed by the post hoc Tukey test (*p* < 0.05).

## 3. Results

The mean and standard deviation of microhardness and surface roughness of the studied groups are presented in Table 1. The one-way ANOVA results revealed significant differences in microhardness and roughness values among the experimental groups (*p*-value < 0.001).

**Figure 4:**
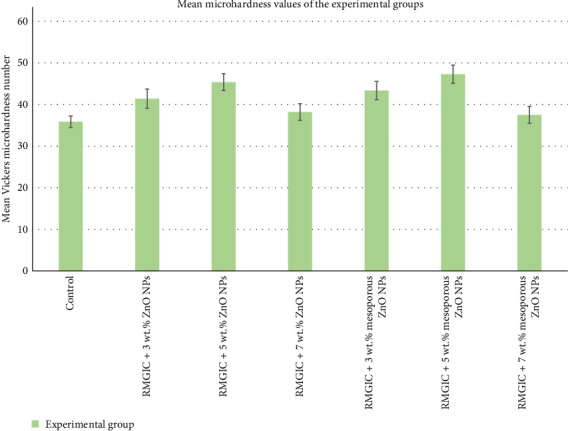
Bar chart of mean microhardness values for all the study groups (*n* = 10 per group). Error bars represent standard deviation. RMGIC, resin-modified glass ionomer cement; ZnO NPs, zinc oxide nanoparticles.

Figure 4 presents a bar chart depicting the microhardness values of the experimental groups. According to post hoc Tukey test, incorporating 5 wt.% of ZnO NPs or mesoporous ZnO NPs into RMGIC resulted in the highest microhardness values (*p*-values < 0.05). The lowest microhardness values were recorded in the control group and in the groups containing 7 wt.% ZnO NPs or mesoporous ZnO NPs (Figure 4), all of which were significantly lower than the values observed in the 3 and 5 wt.% nanoparticle-containing groups (*p*-values < 0.05). Despite this, no significant differences were observed in microhardness values between RMGIC with 3, 5, or 7 wt.% ZnO NPs and their corresponding mesoporous ZnO NP groups (*p*-values > 0.05).

RMGIC with 3 wt.% ZnO NPs and RMGIC with wt. 3 wt.% mesoporous ZnO NPs exhibited significantly higher microhardness values compared to RMGIC with 7 wt.% ZnO NPs and RMGIC with 7 wt.% mesoporous ZnO NPs (*p*-values < 0.05). Therefore, the incorporation of up to 5 wt.% ZnO NPs or mesoporous ZnO NPs into RMGIC led to a significant increase in microhardness values, whereas a 7 wt.% concentration did not demonstrate a significant impact on microhardness values.

**Figure 5:**
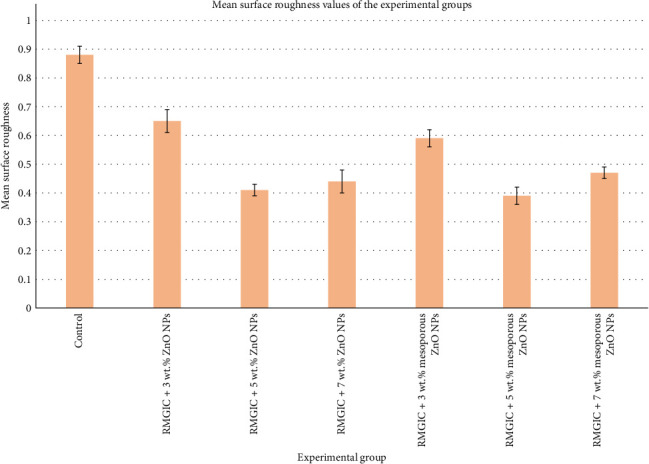
Bar chart of mean surface roughness values for all the study groups (*n* = 10 per group). Error bars represent standard deviation. RMGIC, resin-modified glass ionomer cement; ZnO NPs, zinc oxide nanoparticles.

Figure 5 illustrates a bar graph representing the surface roughness values across the experimental groups. The post hoc Tukey test revealed that the control group exhibited the highest roughness value (*p*-values < 0.05). Following the control group, RMGIC with 3 wt.% ZnO NPs showed higher roughness values compared to other experimental groups (*p*-values < 0.05). Conversely, the lowest roughness values were observed for RMGIC with 5 wt.% mesoporous ZnO NPs followed by RMGIC with 5 wt.% ZnO NPs (*p*-values < 0.05).

There were no significant differences between the roughness values of RMGIC with 5 wt.% ZnO NPs and RMGIC with 5 wt.% mesoporous ZnO NPs, as well as between RMGIC with 5 wt.% ZnO NPs and RMGIC with 7 wt.% ZnO NPs (*p*-values > 0.05). The roughness value of RMGIC with 3 wt.% mesoporous ZnO NPs was statistically higher than that of RMGIC with 7 wt.% ZnO NPs or mesoporous ZnO NPs (*p*-values < 0.05). Conversely, the roughness value of RMGIC with 3 wt.% mesoporous ZnO NPs was statistically lower than that of RMGIC with 3 wt.% ZnO NPs (*p*-value < 0.05). There was no statistical difference in the roughness values between RMGIC with 7 wt.% mesoporous ZnO NPs and RMGIC with 7 wt.% ZnO NPs (*p*-value > 0.05).

The study results indicate that incorporating up to 5 wt.% ZnO NPs or mesoporous ZnO NPs into RMGIC significantly decreased surface roughness. However, increasing the concentration to 7 wt.% resulted in increased roughness values. Even with the addition of up to 7 wt.% NPs into RMGIC, lower roughness values were observed compared to the control group.

## 4. Discussion

The results of this study led to the rejection of the null hypothesis as notable variances in microhardness and surface roughness were evident when comparing RMGIC samples with and without the incorporation of mesoporous ZnO NPs and ZnO NPs. The study findings indicated that incorporating 5 wt.% ZnO NPs or mesoporous ZnO NPs into RMGIC resulted in peak microhardness values, while excessive concentrations led to diminished outcomes. Concentrations up to 5 wt.% notably improved microhardness and reduced roughness, showcasing an optimal range for enhancing material characteristics.

Enhancing the bonding performance and structural integrity of dental restorative materials is essential for ensuring their long-term clinical success, particularly in challenging oral environments where mechanical stress, moisture, and biofilm accumulation can compromise restoration durability [24–26]. This is also relevant for GICs, which, despite their favorable properties such as chemical adhesion and fluoride release, may exhibit limitations in mechanical strength and wear resistance [7, 8, 24].

Dental caries primarily stem from cariogenic bacteria, notably *Streptococcus mutans*, which play a pivotal role in biofilm formation and subsequent caries. Even with meticulous caries removal, bacteria are often not entirely eradicated from prepared tooth surfaces. Moreover, gaps forming at the junction between the tooth and the restorative material like GIC can facilitate bacterial infiltration, potentially resulting in secondary caries formation [15, 27].

GICs exhibit antibacterial properties primarily due to fluoride release, although some studies attribute this effect to the low pH during setting [27]. However, their antibacterial activity postsetting remains uncertain, and like composites, secondary caries is a major cause of failure, suggesting fluoride release alone may be insufficient for complete bacterial suppression [27]. Developing antibacterial-modified GICs is essential to reduce biofilm-associated caries and improve restoration longevity. Therefore, various antibacterial agents—such as quaternary ammonium methacrylates, epigallocatechin-3-gallate, propolis extracts, and chlorhexidine (CHX)—have been incorporated to enhance GICs' antimicrobial potential [3, 14, 27, 28].

Metal and metal oxide NPs are promising additives in restorative dentistry due to their biocompatibility, high bioactivity, large surface-to-volume ratio, and favorable mechanical properties [14, 15, 27]. Their integration into dental materials enhances both mechanical strength and antibacterial efficacy, with minimal side effects such as allergic reactions [14, 18, 29].

ZnO NPs have gained prominence in dental research for their strong antibacterial activity, which is closely linked to their high surface area [15]. They are also valued for their safety, stability, biocompatibility, low toxicity, and affordability [15]. Studies show that ZnO NPs reduce biofilm formation by up to 85% and exhibit significant antibacterial effects against *S. mutans* and *Lactobacillus* when incorporated into resin composites [30, 31]. Additionally, ZnO NPs can enhance the antibacterial, mechanical, and bonding properties of resin composites [18].

Mesoporous materials have recently gained attention in dentistry due to their high surface area, tunable porosity, and controlled drug delivery potential [32]. Mesoporous calcium–silicate NPs loaded with CHX have shown strong antibacterial activity against *E. faecalis*, low cytotoxicity, and ion release properties, making them suitable for bone repair and intracanal applications [32]. Similarly, mesoporous silica NPs (MS NPs) have enabled sustained CHX release from resin composites, inhibiting *S. mutans* and *L. casei* without compromising mechanical integrity [3, 20]. Mesoporous ZnO NPs—previously proposed for ureteral stents due to their biodegradability and antibacterial properties—also show promise in dentistry as bioactive fillers [33]. One study demonstrated that incorporating Zn-MSNs into dental resin increased antibacterial efficacy up to 100% at 15 wt.% without affecting the depth of cure or cytocompatibility [34]. Therefore, mesoporous ZnO NPs were selected in this study for incorporation into RMGIC due to their antibacterial properties and high surface area, which may offer advantages over conventional ZnO NPs. However, their effects on key mechanical properties, such as surface roughness and microhardness, must be clarified before clinical application. ZnO and mesoporous ZnO NPs were chosen in this study for their strong affinity with the polyacrylic matrix of RMGIC and documented antibacterial activity [35, 36]. Therefore, this study aimed to compare the effects of both nanoparticle types on RMGIC's surface properties while preserving its mechanical integrity.

While incorporating NPs into restorative materials can reduce bacterial adhesion, their addition must not compromise mechanical properties. Inadequate surface hardness and roughness can reduce material strength, increase water absorption, and undermine the durability of restorations [37]. Surface hardness is essential for resisting deformation, while lower microhardness may accelerate degradation, promote plaque accumulation, and alter surface characteristics during clinical use [9, 37]. Surface roughness also affects wear and marginal adaptation, necessitating accurate clinical evaluation [7, 9, 37]. This study employed profilometry for roughness assessment due to its precision and usability, with polishing performed beforehand to simulate clinical conditions [9, 38]. Filler particle size and content significantly influence hardness and roughness—smaller particles enhance polishability, while larger or irregular fillers increase surface roughness [39, 40]. Current GIC formulations, including glass hybrid and high-viscosity types, often fall short of clinical expectations, reinforcing the need to improve their surface properties [7].

According to the present findings, incorporating up to 5 wt.% mesoporous ZnO NPs into RMGIC significantly improved both microhardness and surface roughness, underscoring the influence of nanoparticle structure on material performance. This enhancement may be explained by multiple mechanisms. First, the small particle size of ZnO and mesoporous ZnO NPs allows them to occupy the interstitial spaces between glass fillers and the matrix, contributing to a more compact and uniform structure with improved mechanical integrity. Second, their high surface area—especially in the mesoporous form—increases contact with the resin matrix, promoting better interfacial bonding. Additionally, the ordered porous structure of mesoporous ZnO NPs may allow the resin matrix to penetrate their surface channels, forming micromechanical interlocks that further enhance hardness and reduce surface irregularities [41]. These findings align with prior studies showing that mesoporous fillers improve dental composite strength through similar interlocking mechanisms [34]. A possible chemical interaction between ZnO and the polyacrylic matrix may also facilitate more homogeneous dispersion and particle integration. Furthermore, particle size plays a role in polishability and surface finish. Previous research has linked smaller, more uniform particles with smoother surfaces in RMGICs, supporting the observed reduction in surface roughness in our study at 3–5 wt.% NP concentrations [38, 42].

Incorporating 7 wt.% of ZnO or mesoporous ZnO NPs into RMGIC negatively affected microhardness and surface roughness in the present study, likely due to NPs agglomeration. At this concentration, excess NPs may exceed the saturation threshold, leading to poor dispersion, particle clustering, and disruption of matrix–filler bonding. These agglomerates can create weak zones, reduce reinforcement efficiency, and increase surface irregularities. Horszczaruk et al. [43] similarly reported that NP aggregation weakens cement structures by limiting uniform integration. Moreover, high NP loading may lower the relative Al^3+^ content, which is essential for polyacrylic acid crosslinking and material strength [43, 44]. The synthetic nature and lack of functional groups in some NPs may further hinder chemical compatibility with the RMGIC matrix. To optimize performance, NP concentrations should be carefully controlled, and future research should explore surface-functionalized mesoporous ZnO NPs to improve matrix interaction.

Mesoporous ZnO NPs were evaluated as reinforcement agents in RMGIC alongside conventional ZnO NPs. To the authors' knowledge, this was the first assessment of their kind in this context. Incorporating up to 5 wt.% improved microhardness without adversely affecting surface roughness, underscoring the importance of NP structure and concentration. These results support the potential of mesoporous ZnO NPs to enhance dental restorative materials. Future studies should explore their effects on additional mechanical properties to confirm and extend these findings.

One limitation of this study is its in vitro design, which does not replicate clinical conditions such as oral acidity, chewing, and brushing forces. As a result, the findings may not fully represent the performance of RMGICs containing mesoporous ZnO and ZnO NPs in the oral environment. Future studies should investigate a wider range of NP concentrations and evaluate additional mechanical and physical properties to better assess their clinical applicability and overall effectiveness.

## 5. Conclusion

In conclusion, this study demonstrated that incorporating up to 5 wt.% of mesoporous ZnO NPs or ZnO NPs into RMGIC significantly improved microhardness while reducing surface roughness. Higher concentrations (e.g., 7 wt.%) led to diminished mechanical performance, likely due to NP agglomeration and suboptimal dispersion. These findings highlight the potential of mesoporous ZnO NPs to enhance both the mechanical and antibacterial properties of RMGIC. This study is among the first to evaluate mesoporous ZnO NPs as reinforcement agents in RMGIC, and future research should include clinical assessments to validate these results and explore additional mechanical and functional outcomes.

## Figures and Tables

**Table 1:** Mean (standard deviation) of microhardness and surface roughness across the study groups (*n* = 10 per group).

Group number	Group description	Microhardness (VHN)	Roughness (µm)
1	Control	35.88 ± 1.38^A^	0.88 ± 0.03^A^
2	RMGIC + 3 wt.% ZnO NPs	41.41 ± 2.32^BC^	0.65 ± 0.04^B^
3	RMGIC + 5 wt.% ZnO NPs	45.38 ± 2.02^CD^	0.41 ± 0.02^CD^
4	RMGIC + 7 wt.% ZnO NPs	38.21 ± 1.99^AB^	0.44 ± 0.04^CD^
5	RMGIC + 3 wt.% mesoporous ZnO NPs	43.38 ± 2.22^BC^	0.59 ± 0.03^BC^
6	RMGIC + 5 wt.% mesoporous ZnO NPs	47.30 ± 2.18^D^	0.39 ± 0.03^D^
7	RMGIC + 7 wt.% mesoporous ZnO NPs	37.52 ± 2.03^AB^	0.47 ± 0.02^CD^

*Note:* Superscript letters indicate statistical groupings within the same column; values sharing at least one letter are not significantly different, while values with different letters differ significantly (*p* < 0.05).

Abbreviations: RMGIC, resin-modified glass ionomer cement; ZnO NPs, zinc oxide nanoparticles.

## Data Availability

The data are available upon request from the authors.
